# Clinical Care Team’s Guide for Awareness on Risk Assessment of Eltrombopag Complicating Acute Kidney Injury in Relapsed Immune Thrombocytopenic Patients: A Case Report

**DOI:** 10.3390/medicina59091645

**Published:** 2023-09-11

**Authors:** Eman Mostafa Hamed, Mohamed Hussein Meabed, Ahmed R. N. Ibrahim, Ahmed M. Khalaf, Doaa Mohamed El Demerdash, Marwa O. Elgendy, Haitham Saeed, Tamer M. Mahmoud, Heba F. Salem, Hoda Rabea

**Affiliations:** 1 Department of Clinical Pharmacy, Faculty of Pharmacy, Nahda University (NUB), Beni-Suef 62521, Egypt; marwa.elgendy@nub.edu.eg; 2 Department of Pediatrics and Hematology, Faculty of Medicine, Beni-Suef University, Beni-Suef 62521, Egypt; m1hmeabed2@med.bsu.edu.eg; 3 Department of Clinical Pharmacy, College of Pharmacy, King Khalid University, Abha 61421, Saudi Arabia; 4 Department of Internal Medicine and Clinical Hematology, Beni-Suef University, Beni-Suef 62521, Egypt; dr.ahmed201176@yahoo.com; 5 Department of Internal Medicine and Clinical Hematology, Faculty of Medicine, Cairo University, Giza 54212, Egypt; dr_eldemerdash@kasralainy.edu.eg; 6 Department of Clinical Pharmacy, Faculty of Medicine, Beni-Suef University Hospitals, Beni-Suef University, Beni-Suef 62521, Egypt; 7 Clinical Pharmacy Department, Faculty of Pharmacy, Beni-Suef University, Beni-Suef 62521, Egypt; haitham.sd1@gmail.com (H.S.); hoda.ahmed@pharm.bsu.edu.eg (H.R.); 8 Internal Medicine Department, Faculty of Medicine, Beni-Suef University, Beni-Suef 62521, Egypt; drtamer.mohamed@med.bsu.edu.eg; 9 Department of Pharmaceutics and Industrial Pharmacy, Faculty of Pharmacy, Beni-Suef University, Beni-Suef 62521, Egypt; heba.salem@nub.edu.eg; 10 Pharmaceutics and Industrial Pharmacy Department, 6 October Technological University, Giza 62521, Egypt

**Keywords:** autoimmune disease, immune thrombocytopenia, relapsed, acute kidney injury, drug induced, Eltrombopag, corticosteroid resistance

## Abstract

Immune thrombocytopenia (ITP) is an autoimmune bleeding disorder caused by antigen-specific T cells and antiplatelet autoantibodies that inhibit platelet production in the bone marrow or destroy platelets in the spleen. ITP is a form of autoimmunity and is closely associated with inflammation. Corticosteroids are the first-line therapy for ITP, with a total response rate of 53–80%. However, corticosteroid therapy is associated with significant side effects and is often ineffective in patients with corticosteroid-resistant or -intolerant disease. Eltrombopag has been validated as a second-line option in ITP therapy. Despite several studies demonstrating the efficacy and safety of Eltrombopag in immune thrombocytopenia patients, the prevalence of Eltrombopag-induced acute kidney injury has been observed. This case report describes a patient who experienced acute kidney injury during Eltrombopag therapy. A sudden increase in serum creatinine to 6.7 mg/dL and metabolic acidosis occurred after eight weeks of Eltrombopag. The patient’s renal failure had worsened, proteinuria was detected, and emergency hemodialysis was initiated. With vigilant kidney function screening and prompt treatment, the patient’s renal function improved remarkably following cessation of Eltrombopag and initiation of hemodialysis. This case highlights the importance of comprehensive medication history-taking and vigilant kidney function screening in patients receiving Eltrombopag.

## 1. Introduction

Acute kidney injury (AKI) is characterized by an upsurge in serum creatinine (SCR) at least 0.3 mg/dL through 2 days or a 50% increase over 7 days or an eGFR below 60 mL/min/1.73 m^2^ or prolonged oliguria for 6 h [1]. A steady decline in kidney function or ongoing kidney dysfunction associated with an irreversible loss of nephrons and kidney cells can result in chronic kidney disease [2]. Drugs excreted via the kidneys, such as vancomycin, are known to cause drug-induced kidney injury, which can result in the accumulation of the drug and its metabolites, further amplifying kidney toxicity [1].

Autoimmune disease is characterized by self-antigen-induced chronic immune system activation, ultimately leading to tissue inflammation in genetically susceptible individuals. Moreover, inflammation can cause ITP. For instance, serum uric acid, an inflammatory mediator, contributes to the pathophysiology of ITP [3]. Platelets also mediate inflammation and immune-mediated disorders via multiple mechanisms, including the release of pro-inflammatory mediators and surface inflammation-related molecules, and inducing interactions between endothelial cells and leukocytes [4]. Platelets modulate monocyte survival surface molecules following phagocytosis by the system of mononuclear phagocytes [5].

Primary ITP is an autoimmune hematological syndrome categorized by a platelet count of less than 100 × 10^9^/L [6,7]. The pathogenesis of ITP is multifactorial and complicated. The platelet destruction happens through T cell-mediated platelet destruction and/or platelet autoantibodies and/or diminished platelet production by bone marrow-residing megakaryocytes [8]. Patients with immune thrombocytopenia (ITP) are unable to mount a robust TPO-mediated response to compensate for the immune-mediated destruction of platelets, resulting in a net reduction in platelet counts that may cause hemorrhage symptoms [9,10]. Secondary ITP is associated with other conditions, such as drug-induced thrombocytopenia, infections, lymphoproliferative disorders, and rheumatological diseases [11]. ITP patients are at risk of spontaneous bleeding, from petechiae and minor injuries to brain hemorrhage, due to thrombocytopenia [12].

Corticosteroids have been the first-line treatment for ITP since the 1950s, although long-term use can have harmful side effects [13]. Second-line treatments, such as Rituximab and thrombopoietin receptor agonists (TPO-RAs) like Eltrombopag and Romiplostim, are recommended in cases of steroid resistance or intolerance [14]. However, none of these therapies have been universally accepted as the most effective for all ITP patients [15]. Relapsed ITP refers to patients who relapsed following the initial response to first-line corticosteroid therapy and conventional therapies [16].

TPO-RAs are a class of platelet growth factors that replicate the action of endogenous thrombopoietin (TPO) on megakaryocytes and megakaryocyte precursors, thereby promoting their growth and differentiation and augmenting platelet production [17]. Eltrombopag is a non-peptide thrombopoietin receptor agonist that promotes megakaryocyte growth [18]. Recent ITP studies reported that adverse events had happened during Eltrombopag use, such as thrombosis, liver cirrhosis, elevated liver enzymes, osteoporosis, pulmonary hypertension, plantar fasciitis, numbness, tingling, headache, dizziness, and fatigue [19,20,21].

During recent trials, Eltrombopag treatment was withdrawn from one male patient because of stark portal vein thrombosis [22]. Another key adverse event was later documented in a 48-year-old man who started taking Eltrombopag for ITP but subsequently developed a pulmonary embolism and kidney vein occlusion but with no signs of acute kidney injury [23]. However, we reported a severe case of ITP that was worsened by acute kidney injury during Eltrombopag treatment. Only two cases of kidney injury related to Eltrombopag medication have been documented thus far [24,25]. This case report aims to increase clinicians’ awareness of Eltrombopag’s potentially adverse effects and the importance of closely monitoring kidney function in patients who have just started it. To our knowledge, this is the third described case of AKI ascribed to ITP patients commencing Eltrombopag treatment.

## 2. A Case Presentation

**Figure 1:**
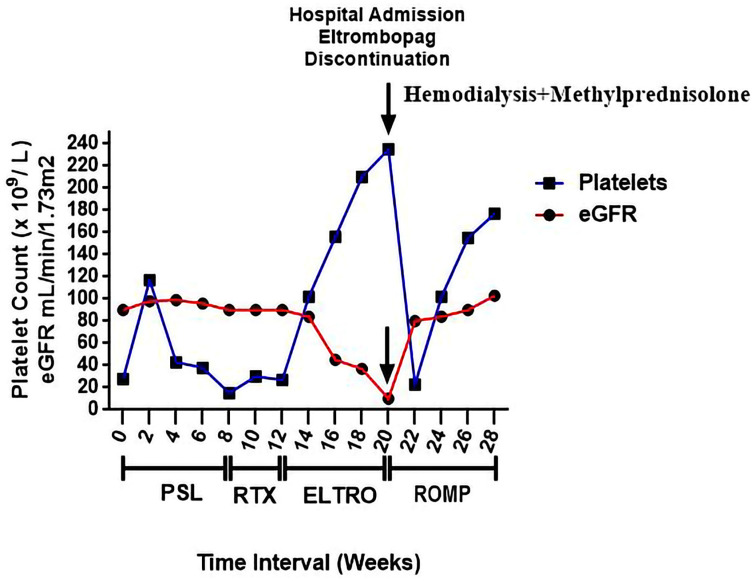
The patient’s glomerular filtration rate and platelet count over time, with drug therapy (including Prednisolone (PSL), Rituximab (RTX), Eltrombopag (ELTRO), and Romiplostim (ROMP)), Eltrombopag discontinuation, and clinical management.

**Figure 2:**
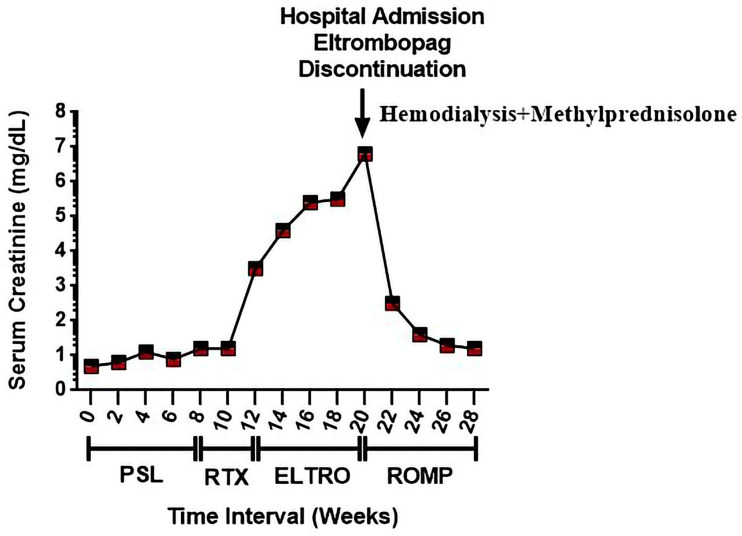
The serum creatinine level over time during Eltrombopag therapy and clinical management with hemodialysis and high dose of Methylprednisolone as shown.

A 45-year-old male patient with relapsed immune thrombocytopenia (ITP) presented with a two-week history of dyspnea, progressive bilateral leg edema, hematuria, persistent nausea, and vomiting. Upon examination, he had bilateral pleural effusions and severe pitting peripheral edema. His kidney biochemistry revealed 3+ proteins in the urine dipstick, an increased serum creatinine level of 6.7 mg/dL, and a decreased eGFR of 10 mL/min/1.73 m^2^ (Figure 1 and Figure 2). Arterial blood gases showed a PaCO_2_ of 13 mmHg, a pH of 7.23, and a HCO_3_ level of 5.3 mmol/L. The patient’s platelet count was 235 × 10^9^/L, and his albumin level was 18 g/L (normal: 35–50 g/L). The protein-to-creatinine ratio in his urine was 1041 mg/mol (normal: 35 mg/mol). The patient was diagnosed with acute kidney injury and interstitial nephritis. The ethical approval committee of the Faculty of Pharmacy at Beni-Suef University provided approval for the case report, with the registration number REC-H-PhBSU-22016. To raise awareness of this adverse event, we have registered it in the pharmacovigilance of the Egyptian Drug Authority with the number 11-332-024-645.

### 2.1. Patient’s Past History

The patient initially presented to our hematologic clinic with purpura, epistaxis, and a platelet count of 28 × 10^9^/L. ITP was diagnosed, and a high dose of Prednisolone was started at 20 mg three times daily [15,26]. Two weeks later, the PLT improved to 117 × 10^9^/L, and the same Prednisolone dose was continued for a week and then tapered to 10 mg twice daily. Two weeks later, the platelet count dropped to 43 × 10^9^/L, and the Prednisolone dose was increased to 40 mg twice daily. The platelet count was recovered to 50 × 10^9^/L but dropped to 38 × 10^9^/L a month later and then to 15 × 10^9^/L, with no response to Prednisolone dose modifications. The patient’s Prednisolone doses were adjusted to 5–10 mg daily, and the platelet count remained in the range of 10–35 × 10^9^/L. The patient discontinued the corticosteroid because of adverse events and loss of response. Meanwhile, the patient had gastroesophageal reflux disease and received 40 mg of oral pantoprazole once daily for 5 days. He also received celecoxib for 3 days for pain relief for a spinal disc herniation.

The patient responded poorly to the standard dose and high dose of Prednisolone. Rituximab at a dose of 375 mg/m^2^ (500 mg) for four weeks was started [27], but the platelet count remained low at 31 and 27 × 10^9^/L. After discussion with the patient, Eltrombopag was chosen and started orally at 12.5 mg daily instead of a splenectomy. The Eltrombopag dose was gradually increased to 50 mg in the second week [28] and increased the platelet count from 15 × 10^9^/L to 156 × 10^9^/L during the first two weeks. At the start of Eltrombopag treatment, the patient had an estimated glomerular filtration rate (eGFR) of 90 mL/min/1.73 m^2^. Eltrombopag treatment successfully increased the platelet count to 235 × 10^9^/L over the first eight weeks but the patient developed severe acute kidney injury (AKI).

After eight weeks of Eltrombopag treatment, the patient was admitted to the emergency unit for acute kidney injury. The Eltrombopag treatment was stopped. This patient was unresponsive to corticosteroids and was switched to Romiplostim at a dose of 3 μg/kg, which was subcutaneously injected weekly to manage chronic ITP [29]. The patient’s platelet count returned to 102 × 10^9^/L and hematocrit to 33.9% after two weeks of Romiplostim therapy, as shown in Figure 1.

### 2.2. Laboratory Examinations

Unremarkable results were obtained from a primary screening using biochemistry tests and autoimmune profiles. Antinuclear antibodies, anti-neutrophilic cytoplasmic antibodies, anti-glomerular basement membrane antibodies, and anti-phospholipase 2 receptor antibodies were all negative in the auto-antibody screen. The COVID-19 PCR test also was negative. Medication history did not reveal any further nephrotoxic drug. An ultrasound of the kidneys revealed that both were normal in size and form, with no signs of occlusion.

Besides overall fatigue and poor physical illness, the patient’s liver function tests showed an aspartate aminotransferase level of 125 (normal range: 8–38) U/L, alanine aminotransferase level of 160 (4–44) U/L, lactate dehydrogenase level of 354 (120–245) U/L, alkaline phosphatase level of 531 (105–330) U/L, and γ-glutamyltransferase level of 288 (<80) U/L. A complete blood count (CBC) demonstrated a white blood cell count of 17.2 × 10^9^/L, with a segmented neutrophil level of 81% (normal range 45–75%), and lymphocyte level of 13% (20–45%). The hematocrit was 41.9%, and the platelet count was 235 × 10^9^/L. The serum albumin level was low at 18 gm/L (35–50 gm/L). Upon Eltrombopag discontinuation, the platelet count dropped to 23 × 10^9^/L. Because the patient’s platelet count was continuously below the standard range of 150–400 × 10^9^/L, a kidney biopsy was not performed due to an elevated risk of bleeding. Urinalysis revealed microscopic sediments of 8–15 red blood cells (RBCs), and the serum H. pylori IgG test was negative. The renal duplex scanning revealed no thrombosis in the renal or other abdominal vessels, with no evidence of renal artery stenosis, with normal intra-renal flow, and with patent renal veins.

The laboratory findings suggest that the patient had impaired kidney function, with markedly elevated levels of serum creatinine and BUN, and electrolyte imbalances. The CBC showed leukocytosis with a predominance of segmented neutrophils, consistent with an acute inflammatory response. Urinalysis showed microscopic sediments of RBCs, which could indicate kidney involvement. These laboratory results are profiled in Table 1.

## 3. Clinical Management

The patient has been diagnosed with drug-induced acute kidney injury concomitant with acute liver dysfunction. The patient was admitted to the hospital at the emergency unit. The patient’s kidney damage progressed on day 2 with a uric acid level of 13.7 mg/dL (standard reference 1.8–6.2 mg/dL), SCR level of 7.3 mg/dL, and BUN level of 62 mg/dL. The urine creatinine and protein levels were 43.6 mg/dL and 99.4 mg/dL, respectively, resulting in a random urine protein/urine creatinine ratio of 2.33 (normal; 0.2 or less). Eltrombopag treatment was discontinued due to a correlation between its use and the onset of proteinuria and subsequent kidney damage. The patient also received a 150 mEq 8.4% bicarbonate bolus to control the metabolic acidosis. On day 2, hemodialysis was induced immediately for eight sessions for 2 weeks to control the metabolic acidosis. In addition, the patient received 250 mg of ursodeoxycholic acid every 6 h and 180 mg of Silymarin every 12 h to manage his acute liver injury. The patient was also diagnosed with interstitial nephritis. Therefore, the patient received 500 mg of Methylprednisolone daily for 3 days, then continued with Prednisolone at 60 mg daily for 4 weeks, which was then tapered and discontinued [30,31], and furosemide (0.5 μg/kg/min) to manage the edema [32]. The immediate laboratory investigations following the controlling treatment revealed an HCO_3_ level of 18 mmol/L, pH of 7.44, sodium level of 134 mmol/L, potassium level of 3.9 mmol/L, and urea level of 12.4 mmol/L. This patient was discharged. His eGFR increased to 80 mL/min/1.73 m^2^, then to 84, 90, and 103 mL/min/1.73 m^2^, and his urine polymerase chain reaction (PCR) test decreased to 103 mg/mmol due to the remarkable response. In addition, his BUN, serum albumin, and serum creatinine levels were recovered to 14 mg/dL, 43 gm/L, and 1.2 mg/dL, respectively. The patient’s liver function tests were improved upon discontinuing Eltrombopag treatment and receiving liver supplements. His aspartate aminotransferase level decreased to 32 (normal range: 8–38) U/L, alanine aminotransferase level to 39 (4–44) U/L, lactate dehydrogenase level to 214 (120–245) U/L, alkaline phosphatase level to 288 (105–330) U/L, and γ-glutamyltransferase level to 67 (<80) U/L. As observed, the patient was effectively weaned off the corticosteroids by week 24, and his kidney function had recovered to the optimal level (Figure 2). The patient experienced severe thrombocytopenia and progressive hypochromic anemia during the treatment, which was managed with supportive care and close monitoring. The patient’s platelet count increased to 155 × 10^9^/L during Romiplostim therapy, which stabilized over the next 4 weeks (>160 × 10^9^/L).

Finally, the clinical management plan for the patient with drug-induced acute renal injury was based on the patient’s clinical presentation and laboratory findings. The Eltrombopag discontinuation and use of bicarbonate bolus, hemodialysis, high-dose Methylprednisolone, and furosemide effectively improved the patient’s kidney function and controlled the metabolic acidosis. The patient’s response to treatment was closely monitored, and adjustments were made as necessary. The patient’s remarkable response to treatment highlights the importance of early recognition and prompt management of drug-induced kidney injury.

## 4. Discussion

This case report documented an acute kidney injury observed in an immune thrombocytopenia patient receiving Eltrombopag therapy. The patient’s kidney function remarkably improved after discontinuing Eltrombopag therapy and receiving hemodialysis.

Eltrombopag is an effective and safe therapeutic choice in cases of corticosteroid-resistant or relapsed ITP [23]. There is an intensified risk of AKI associated with Eltrombopag use. Although the Phase I and Phase II studies of Eltrombopag have revealed non-serious symptoms, such as arthralgia, influenza-like illness, constipation, myalgia, diarrhea, and pruritus, with no reports of AKI [33], this case report documented one episode of AKI associated with Eltrombopag therapy. Nephrotic syndrome and AKI are reported as adverse events of Eltrombopag therapy in humans [25]. Kidney injury has been observed in animal studies [33], but two studies reported this issue. In the first case report, one episode of acute kidney injury was reported in a recently published trial during Eltrombopag treatment. This case report reported a 77-year-old ITP patient with a history of hypertension and two risk factors that may have contributed to the development of nephrotic syndrome [25]. In the current case report, the patient had no risk factors related to age or other comorbidities. A second case report also documented two episodes. In the first episode, a 54-year-old man with ITP and antiphospholipid syndrome developed AKI during Eltrombopag therapy. A kidney biopsy was not performed because kidney function returned to baseline when the Eltrombopag therapy was discontinued. In the second episode, after receiving seven doses of Eltrombopag therapy for ITP, the patient experienced AKI and nephrotic syndrome. A kidney biopsy revealed focal segmental glomerulosclerosis (FSGS) upon kidney biopsy examination [24].

The potential mechanisms of Eltrombopag-associated kidney dysfunction remain speculative. One hypothesis suggests that Eltrombopag may trigger the activation of cytoplasmic tyrosine kinases in megakaryocytes, which in turn activates signal transducers and activators of phosphoinositide-3, kinase transcription 5, and Ras-mitogen-activated protein kinase (MAPK) [34]. In rat models, the activated megakaryocytes release transforming growth factor-beta [25,35], which activates MAPK-ERK and PI3K pathways in podocytes and results in podocyte apoptosis and the progression of glomerulosclerosis with proteinuria [35].

Another theory explains the possible mechanisms of Eltrombopag-induced kidney injury. The kidney has metabolically active enzyme systems that activate medicines and other substances. Because of this, a thorough examination of the potential contribution of metabolic activation to the pathogenesis of many drug-induced kidney disorders has been undertaken. Approximately 30% of the Eltrombopag is excreted by the kidneys after hepatic metabolism [36]. The thrombopoietin receptor is not found in normal kidney tissue, but it is thought to be expressed in the bone marrow and confined to CD34+ cells, megakaryocytes, platelets, and endothelial and dendritic cells [37,38]. However, reverse transcriptase-polymerase chain reaction tests can detect a very small amount of mRNA in human kidney carcinoma cell lines [18].

Although the patient was taking pantoprazole and celecoxib, a review of the patient’s drug history revealed that these medications were unlikely to be the cause of kidney injury. Celecoxib was used for only 3 days, and the interval between the discontinuation of celecoxib and the development of kidney injury (>3 months) greatly exceeded its half-life (11 h). Furthermore, a normal serum creatinine concentration was documented during the pantoprazole use. An elevation in the serum creatinine concentration generally starts after 4 months of discontinuation of pantoprazole. Therefore, there is no mention of concomitant medication-induced kidney injury.

Using the Naranjo adverse drug reaction probability scale [39] from the questionnaire scale, we confirmed that the patient’s development of AKI and use of Eltrombopag medication were likely related (score of 7). The Naranjo scale has been applied in case reports and series of overdose patients to evaluate the probability of an adverse event caused by the oral drug or therapeutic modality [39].

There was a mild elevation in the liver enzymes but not so severe to the extent that this change led to kidney disease, suggesting that the liver injury was not severe [40,41]. This study confirmed that the acute liver dysfunction was reversed after Eltrombopag discontinuation and liver supplement use.

The absence of alternative causes of kidney injury and the similarity to the prior two case reports [24,25] support our inference. Notably, the patient’s kidney function improved after the discontinuation of Eltrombopag and treatment with bicarbonate and steroids, requiring kidney replacement therapy.

The absence of other possible causes and reports of similar clinical presentations support our inference. The European Medicines Agency’s product information for Eltrombopag mentions renal impairment as an uncommon adverse effect [24,25,33]. The incidence of proteinuria and serum creatinine elevation was considered as a common side effect in the most recent summary of the product characteristics of Eltrombopag [25,42,43,44]. Renal failure, nephritis, nocturia, blood urea elevation, and urine protein-to-creatinine ratio elevation were documented as uncommon adverse drug reactions in ITP patients treated with Eltrombopag [25,42,43,44]. Notably, Eltrombopag pharmacokinetics were altered in kidney and liver dysfunction cases [24,25]. Multiple studies have suggested that Eltrombopag should be used with caution in renal impairment patients [33,42,43,44,45,46]. There are proteinuria manifestations that confirm the existence of Eltrombopag-induced renal injury with persistent use [35]. Moreover, there are no related risk factors, including pre-renal, post renal, and intra-renal factors, with no medication- or disease-induced kidney injury. This subtlety further emphasizes the need for enhanced clinical vigilance when using Eltrombopag, especially in patients with renal impairment or who have risk factors.

This patient was unresponsive and intolerant to corticosteroids, Eltrombopag, and Rituximab. The patient had severe acute kidney injury during Eltrombopag treatment. Therefore, this reinforces the idea that, even though newer lines of therapy have evolved and become more widely used in recent years, splenectomy remains a safe option in cases of conventional therapy relapsed ITP.

Through this case, the aim is to inform clinicians about Eltrombopag’s potential adverse events and the need for closer monitoring of kidney function in patients who are beginning Eltrombopag treatment. These observations also might suggest the future prerequisite for sequential clinical trials. Finally, recognition of Eltrombopag-induced nephropathy requires a comprehensive medication history and consideration of the drug in the differential diagnosis of patients presenting with acute kidney injury and concomitant hepatic dysfunction, and an understanding of the possible risk factors that may reduce the drug’s margin of safety at therapeutic doses. This case highlights the importance of comprehensive medication history-taking and vigilant kidney function screening in patients receiving Eltrombopag.

## 5. Conclusions

A temporal relationship was observed between AKI and the administration of Eltrombopag, with improvements in renal function after discontinuing the medication. It may be prudent for clinicians to monitor kidney function during Eltrombopag therapy. This case serves as an example of the need for a clinical pharmacist with training to handle drug-related issues in a hospital setting to prevent future neglect. This is because the presence of the clinical pharmacist yields case awareness and regular kidney function follow-up at the start of therapy in the outpatient care setting. Clinicians should be vigilant about the potential association between Eltrombopag and kidney injury, and close monitor kidney function in patients receiving this medication.

### Case Report Limitation

Further research is necessary to investigate Eltrombopag-associated kidney dysfunction’s underlying mechanisms and identify alternative treatment options for patients with relapsed ITP.

## Figures and Tables

**Table 1:** The Serological investigations detected during patient admission.

Serological Test	Result	Reference Range
White blood cell count	17.2	4–11 × 10^9^/L
Platelet count	235 × 10^9^/L	150–400 × 10^9^/L
Serum creatinine	6.7	0.8–1.4 mg/dL
Serum potassium	5.6	3.4–4.7 mEq/L
Serum albumin	18	35–50 g/L
γ-glutamyltransferase	188 U/L	(5–40) U/L
Serum lactate dehydrogenase	354	(105–333) U/L
Antinuclear antibodies	Negative (0.5)	(0.0–1.0)
Anti-neutrophilic cytoplasmic antibodies	Negative	Negative
Serum protein electrophoresis	Negative for Paraproteins	Negative
Anti-glomerular basement membrane antibodies	Negative < 3.1 Elia U/mL	(0.0–10.0 Elia U/mL)
Anti-phospholipase 2 receptor antibody	Negative (<4.6)	<15
Serum IgA	1.34 g/L	(0.8–4.0 g/L)
Serum IgM	0.81 g/L	(0.94 g/L) (0.5–2.0 g/L)
Serum IgG	9.8 g/L	(6.0–16.0 g/L)
Complement C3	1.09 g/L	(0.75–1.65 g/L)
Complement C4	0.36 g/L	(0.14–0.54 g/L)
Rheumatoid factor	17 IU/mL	<30 IU/mL
Anti-double-stranded DNA (IU/mL)	15 IU/mL	<30 IU/mL
Anti-_2-glycoprotein IgG	<20 IU/mL	11 IU/mL
Antiphospholipid IgG	<15 U/mL	6 U/mL
C-ANCA	<7 U/mL	1.1 U/mL
P-ANCA	<7 U/mL	0.7 U/mL
Anti-GBM	<7 U/mL	0.9 U/mL
Cryoglobulin	Negative	Negative
Hepatitis B surface antigen	Negative	Negative
Hepatitis C antibodies	Negative	Negative
HIV 1 and 2	Not detected	Negative
SARS-CoV-2 PCR	Negative	Negative
Erythrocyte sedimentation (ESR)	59 mm/h	0 to 22 mm/h

P-ANCA = protoplasmic-staining anti-neutrophil cytoplasmic antibody; C-ANCA = classic anti-neutrophil cytoplasmic antibody; GBM = glomerular basement membrane; Ig = immunoglobulin.

## Data Availability

The raw data is enclosed within the article.

## References

[1] Kellum J.A., Romagnani P., Ashuntantang G., Ronco C., Zarbock A., Anders H.J. (2021). Acute kidney injury. Nat. Rev. Dis. Primers.

[2] Yerramilli M., Farace G., Quinn J., Yerramilli M. (2016). Kidney disease and the nexus of chronic kidney disease and acute kidney injury: The role of novel biomarkers as early and accurate diagnostics. Vet. Clin. Small Anim. Pract..

[3] Dal M.S., Karakus A., Aydin B.B., Ekmen M.O., Ulas T., Ayyildiz O. (2015). Serum uric acid and inflammation in patients with immune thrombocytopenic purpura: Preliminary results. Eur. Rev. Med. Pharmacol. Sci..

[4] Lugus J.J., Park C., Ma Y.D., Choi K. (2009). Both primitive and definitive blood cells are derived from Flk-1+ mesoderm. Blood.

[5] Lang D., Dohle F., Terstesse M., Bangen P., August C., Pauels H.-G., Heidenreich S. (2002). Down-regulation of monocyte apoptosis by phagocytosis of platelets: Involvement of a caspase-9, caspase-3, and heat shock protein 70-dependent pathway. J. Immunol..

[6] Song F., Al-Samkari H. (2021). Management of adult patients with Immune Thrombocytopenia (ITP): A review on current guidance and experience from clinical practice. J. Blood Med..

[7] Ayad N., Grace R.F., Al-Samkari H. (2021). Thrombopoietin receptor agonists and rituximab for treatment of pediatric immune thrombocytopenia: A systematic review and meta-analysis of prospective clinical trials. Pediatr. Blood Cancer.

[8] Semple J.W., Rebetz J., Maouia A., Kapur R. (2020). An update on the pathophysiology of immune thrombocytopenia. Curr. Opin. Hematol..

[9] González-Porras J.R., Godeau B., Carpenedo M. (2019). Switching thrombopoietin receptor agonist treatments in patients with primary immune thrombocytopenia. Ther. Adv. Hematol..

[10] Rodeghiero F., Stasi R., Gernsheimer T., Michel M., Provan D., Arnold D.M., Bussel J.B., Cines D.B., Chong B.H., Cooper N. (2009). Standardization of terminology, definitions and outcome criteria in immune thrombocytopenic purpura of adults and children: Report from an international working group. Blood.

[11] Kado R., McCune W.J. (2019). Treatment of primary and secondary immune thrombocytopenia. Curr. Opin. Rheumatol..

[12] Kaur M.N., Arnold D.M., Heddle N.M., Cook R.J., Hsia C.C., Blostein M., Jamula E., Sholzberg M., Lin Y., Kassis J. (2022). Cost-effectiveness of Eltrombopag vs intravenous immunoglobulin for the perioperative management of immune thrombocytopenia. Blood Adv..

[13] Provan D., Arnold D.M., Bussel J.B., Chong B.H., Cooper N., Gernsheimer T., Ghanima W., Godeau B., González-López T.J., Grainger J. (2019). Updated international consensus report on the investigation and management of primary immune thrombocytopenia. Blood Adv..

[14] Hamed E.M., Ibrahim A.R., Meabed M.H., Khalaf A.M., El Demerdash D.M., Elgendy M.O., Saeed H., Salem H.F., Rabea H. (2023). Therapeutic Outcomes of High Dose-Dexamethasone versus Prednisolone+ Azathioprine, Rituximab, Eltrombopag, and Romiplostim Strategies in Persistent, Chronic, Refractory, and Relapsed Immune Thrombocytopenia Patients. Pharmaceuticals.

[15] Yasser A., Khasahba E.O., Shokeir M.A.E.R., El Mabood S.A. (2020). Treatment lines of childhood chronic ITP: A retrospective single-center analysis. Вoпрoсы гематoлoгии/oнкoлoгии и иммунoпатoлoгии в педиатрии.

[16] Cuker A., Neunert C.E. (2016). How I treat refractory immune thrombocytopenia. Blood J. Am. Soc. Hematol..

[17] Kuter D.J., Gernsheimer T.B. (2009). Thrombopoietin and Platelet Production in Chronic Immune Thrombocytopenia. Hematol. Clin. N. Am..

[18] Sperati C.J., Streiff M.B. (2010). Acute renal failure in a patient with antiphospholipid syndrome and immune thrombocytopenic purpura treated with Eltrombopag. Am. J. Hematol..

[19] Hamed E.M., Ibrahim A.R.N., Meabed M.H., Khalaf A.M., El Demerdash D.M., Elgendy M.O., Saeed H., Salem H.F., Rabea H. (2023). The Outcomes and Adverse Drug Patterns of Immunomodulators and Thrombopoietin Receptor Agonists in Primary Immune Thrombocytopenia Egyptian Patients with Hemorrhage Comorbidity. Pharmaceuticals.

[20] Mishra K., Pramanik S., Jandial A., Sahu K.K., Sandal R., Ahuja A., Yanamandra U., Kumar R., Kapoor R., Verma T. (2020). Real-world experience of Eltrombopag in immune thrombocytopenia. Am. J. Blood Res..

[21] Giordano P., Lassandro G., Barone A., Cesaro S., Fotzi I., Giona F., Ladogana S., Miano M., Marzollo A., Nardi M. (2020). Use of Eltrombopag in children with chronic immune thrombocytopenia (ITP): A real life retrospective multicenter experience of the Italian Association of Pediatric Hematology and Oncology (AIEOP). Front. Med..

[22] Saito M., Morioka M., Izumiyama K., Mori A., Kondo T. (2021). Severe Portal Vein Thrombosis During Eltrombopag Treatment Concomitant Splenectomy for Immune Thrombocytopenia. Cureus.

[23] Wu C., Zhou X.-M., Liu X.-D. (2021). Eltrombopag-related renal vein thromboembolism in a patient with immune thrombocytopenia: A case report. World J. Clin. Cases.

[24] Teng C.-J., Hong Y.-C., Chiang H.-L., Liu H.-T., Liu C.-J., Yang A.-H., Liu J.-H., Wang W.-S., Tzeng C.-H. (2011). Focal segmental glomerulosclerosis with acute renal failure associated with Eltrombopag therapy. Pharmacother. J. Hum. Pharmacol. Drug Ther..

[25] Ghosh S.A., Patrick J., Maw K.Z. (2021). Acute kidney injury and nephrotic syndrome associated with Eltrombopag therapy in chronic idiopathic thrombocytopenic purpura. BMJ Case Rep..

[26] Neunert C., Terrell D.R., Arnold D.M., Buchanan G., Cines D.B., Cooper N., Cuker A., Despotovic J.M., George J.N., Grace R.F. (2019). American Society of Hematology 2019 guidelines for immune thrombocytopenia. Blood Adv..

[27] Tavakolpour S., Aryanian Z., Seirafianpour F., Dodangeh M., Etesami I., Daneshpazhooh M., Balighi K., Mahmoudi H., Goodarzi A. (2021). A systematic review on efficacy, safety, and treatment-durability of low-dose rituximab for the treatment of Pemphigus: Special focus on COVID-19 pandemic concerns. Immunopharmacol. Immunotoxicol..

[28] Visco C., Rodeghiero F., Romano A., Valeri F., Merli M., Quaresimini G., Volpetti S., Santi R.M., Carli G., Lucchini E. (2019). Eltrombopag for immune thrombocytopenia secondary to chronic lymphoproliferative disorders: A phase 2 multicenter study. Blood.

[29] Puavilai T., Thadanipon K., Rattanasiri S., Ingsathit A., McEvoy M., Attia J., Thakkinstian A. (2019). Treatment efficacy for adult persistent immune thrombocytopenia: A systematic review and network meta-analysis. Br. J. Haematol..

[30] Hodson E.M., Hahn D., Craig J.C. (2015). Corticosteroids for the initial episode of steroid-sensitive nephrotic syndrome. Pediatr. Nephrol..

[31] Seethapathy H., Lee M.D., Strohbehn A.I., Efe O., Rusibamayila N., Chute D.F., Colvin R.B., Rosales A.I., Fadden R.M., Reynolds K.L. (2022). Clinical features of acute kidney injury in patients receiving dabrafenib and trametinib. Nephrol. Dial. Transpl..

[32] Patschan D., Patschan S., Buschmann I., Ritter O. (2019). Loop diuretics in acute kidney injury prevention, therapy, and risk stratification. Kidney Blood Press. Res..

[33] Nieto M., Calvo G., Hudson I., Feldschreiber P., Brown D., Lee C.C., Lay G., Valeri A., Abadie E., Thomas A. (2011). The European Medicines Agency review of Eltrombopag (Revolade) for the treatment of adult chronic immune (idiopathic) thrombocytopenic purpura: Summary of the scientific assessment of the Committee for Medicinal Products for Human Use. Haematologica.

[34] Erickson-Miller C.L., Delorme E., Tian S.-S., Hopson C.B., Landis A.J., Valoret E.I., Sellers T.S., Rosen J., Miller S.G., Luengo J.I. (2009). Preclinical activity of Eltrombopag (SB-497115), an oral, nonpeptide thrombopoietin receptor agonist. Stem Cells.

[35] Lee H.S., Song C.Y. (2010). Effects of TGF-β on podocyte growth and disease progression in proliferative podocytopathies. Kidney Blood Press. Res..

[36] Kim T.O., Despotovic J., Lambert M.P. (2018). Eltrombopag for use in children with immune thrombocytopenia. Blood Adv..

[37] Ninos J.M., Jefferies L.C., Cogle C.R., Kerr W.G. (2006). The thrombopoietin receptor, c-Mpl, is a selective surface marker for human hematopoietic stem cells. J. Transl. Med..

[38] Kumamoto T., Azuma E., Tanaka M., Qi J., Hirayama M., Zhang S.-L., Kobayashi M., Iwamoto S., Komada Y., Yamamoto H. (1999). Human dendritic cells express the thrombopoietin receptor, c-Mpl. Br. J. Haematol..

[39] Seger D., Barker K., McNaughton C. (2013). Misuse of the Naranjo adverse drug reaction probability scale in toxicology. Clin. Toxicol..

[40] Kim Y.-K., Lee S.-S., Jeong S.-H., Ahn J.-S., Yang D.-H., Lee J.-J., Kim H.-J. (2015). Efficacy and safety of Eltrombopag in adult refractory immune thrombocytopenia. Blood Res..

[41] Bidika E., Fayyaz H., Salib M., Memon A.N., Gowda A.S., Rallabhandi B., Cancarevic I. (2020). Romiplostim and Eltrombopag in immune thrombocytopenia as a second-line treatment. Cureus.

[42] Burness C.B., Keating G.M., Garnock-Jones K.P. (2016). Eltrombopag: A review in paediatric chronic immune thrombocytopenia. Drugs.

[43] European Medicines Agency Revolade (Eltrombopag), Product Information. https://www.ema.europa.eu/ema/index.jsp?curl=pages/medicines/human/medicines/001110/human_med_001322.jsp&mid=WC0b01ac058001d124.

[44] Bauman J.W., Vincent C.T., Peng B., Wire M.B., Williams D.D., Park J.W. (2011). Effect of hepatic or renal impairment on Eltrombopag pharmacokinetics. J. Clin. Pharmacol..

[45] Garnock-Jones K.P., Keam S.J. (2009). Eltrombopag. Drugs.

[46] Garnock-Jones K.P. (2011). Eltrombopag: A review of its use in treatment-refractory chronic primary immune thrombocytopenia. Drugs.

